# Volumetric Analysis of Perimetry Tests to Guide Central Testing: The Functional Vulnerability Zone

**DOI:** 10.1016/j.xops.2026.101152

**Published:** 2026-03-12

**Authors:** Jack Phu, Henrietta Wang, Michael Kalloniatis

**Affiliations:** 1 School of Optometry and Vision Science, University of New South Wales, Kensington, New South Wales, Australia; 2 University of Houston College of Optometry, Houston, Texas; 3 School of Medicine (Optometry), Deakin University, Waurn Ponds, Victoria, Australia

**Keywords:** Macula, Standard automated perimetry, 10-2, 24-2, Visual fields

## Abstract

**Purpose:**

To use volumetric analysis applied to total deviation (TD) perimetric data to characterize the presence of residual sensitivity measurable using the 10-2 in lieu of the 24-2 test grid in glaucoma (the functional vulnerability zone [FVZ]) and develop a prototype end-user application for clinical guidance.

**Design:**

Cross-sectional study.

**Subjects:**

Six hundred twenty-six pairs of Humphrey Field Analyzer 10-2 Swedish Interactive Thresholding Algorithm (SITA)-Fast and 24-2 SITA-Faster test results of 160 subjects.

**Methods:**

Total deviation values located within 10° from fixation (10-2, 68 locations; 24-2, 12 locations) were extracted. Volumetric analysis was performed to calculate the difference in TD “volume” (10-2 – 24-2) for each pair of results. Then, 24-2 test locations were interpolated across the central 10° to obtain “equivalent” 10-2 test locations used to calculate number of test locations gained (10-2 – 24-2). A positive TD volume difference and positive gain represented the presence of an FVZ. Linear regression and principal components analysis were used to identify 24-2 test features that may predict the presence of an FVZ.

**Main Outcome Measures:**

Volumetric differences in visual field TD as a surrogate for a FVZ.

**Results:**

Visual field global indices and the number of central 24-2 defects predicted TD volume difference (*P* < 0.0001), with 24-2 mean deviation (MD) showing the highest *R*^2^ (0.32). All parameters had low *R*^2^ values when predicting gain. A simplified model comprising 24-2 MD and number of central 24-2 TD probability (TDP) defects (*P* < 0.01) provided a method for clinically identifying the likelihood of an FVZ. Principal components analysis revealed 2 principal components (24-2 global indices and pointwise probability scores) that accounted for 79.7% of the variance, with principal component 1 accounting for 69.3% and comprising 24-2 MD and number of central 24-2 TDP scores at *P* < 2%.

**Conclusions:**

The FVZ offers a data-driven approach to identifying residual dynamic range using the 10-2 test grid. A prototype model for predicting the likelihood of 10-2 utility in progression analysis is proposed.

**Financial Disclosure(s):**

The authors have no proprietary or commercial interest in any materials discussed in this article.

Perimetric testing is a cornerstone of glaucoma care, providing important information on the patient’s functional status.^1^^,^^2^ Glaucoma is characterized by visual field defects that may affect the peripheral or central regions.^3^^,^^4^ Broadly, the goals of perimetry can be divided into the following: detection/screening, confirmation, or monitoring of visual field defects.^1^ The goals depend on the individual patient’s situation, including the stage of disease.

Although it is ideal to assess both central and peripheral visual fields simultaneously,^5^ the clinical conundrum of balancing limited clinical resources with the need to characterize visual field defects has been the subject of ongoing investigation. Clinically, visual field tests can be broadly classified into relatively centrally focused, such as the 10-2 (or equivalent) test grid, or relatively peripherally focused, such as the 24-2 (or equivalent). The 10-2 test grid has a denser test point arrangement, affording greater resolution for characterizing central visual field defects. The 24-2 test uses more widely spaced test locations to cover a larger area of the visual field, with only 12 covering the central 10°. Regarding the relative densities of the test grids, previous literature has debated whether there is a significant role for the 10-2 in detecting additional central defects compared with the 24-2 test grid.^6^, ^7^, ^8^, ^9^, ^10^ In other words, a 24-2 test grid is likely able to reliably predict the presence of a corresponding 10-2 defect, and thus the role of 10-2 testing, with a likely unnecessarily intensive testing approach at the cross-sectional level,^11^ is limited.

Thus, the other clinical question is the role of 10-2 in progression analysis and when to implement testing given a 24-2 test result. Clinical guidelines and previous studies have generally recommended protocoling a 10-2 test in the presence of advanced glaucomatous loss (such as a mean deviation of worse than –12 dB) to better characterize residual vision.^12^, ^13^, ^14^

In our previous work, we proposed the functional vulnerability zone, a volumetric approach to describing the perimetric visual field.^15^ Analogous to the measurement of the penumbra in the ischemic brain, the functional vulnerability zone is a clinical functional measurement derived from perimetric data. The functional vulnerability zone represents the difference in Hill of Vision volume attained using the 24-2 and 10-2 test grids, and is used to compare the dynamic ranges of the respective test grids within the central visual field. A difference 1-way or another, could provide guidance on the appropriateness of deploying the 10-2 test grid as an adjunct to routine testing using the 24-2 test grid for the purposes of progression analysis (and not just for confirmation of central visual field defects).

Our pilot study used sensitivity to characterize the volume of the Hill of Vision and calculate the functional vulnerability zone. Although sensitivity was used as it was an intrasubject difference (rather than a comparison with a normative database), it is possible to use total deviation (TD) as another approach to volumetric analysis to specifically highlight significant areas of deficit. In the present study, we examined the functional vulnerability zone of the central visual field using TD and propose approaches to choosing when to deploy the 10-2 test grid. Using the functional vulnerability zone, we also present a simple graphic user interface (GUI) that uses 24-2 input data to calculate the likelihood of a benefit of the 10-2 test grid.

## Methods

### Ethics Statement

This study was a retrospective study. Ethics approval for the study was provided by the Human Research Ethics Committee of the University of New South Wales (HC210563). The study adhered to the tenets of the Declaration of Helsinki. Subjects provided written informed consent before inclusion in the study.

### Subject Cohort

We reviewed the clinical records of patients seen within the glaucoma service at Centre for Eye Health (University of New South Wales) who have had both 24-2 and 10-2 visual field tests performed at any point during their follow-up from 2019 onward for inclusion as subjects in the present study. Inclusion criteria were: age >18 years, had concurrent 10-2 and 24-2 testing within the same clinical visit, and a complete medical record for review. We acknowledge issues pertaining to using automated reliability indices (especially false-positive rate)^16^^,^^17^ to evaluate visual field utility, but for consistency with current clinical deployment, we used a false-positive rate of ≤15% as another inclusion criterion.

Although the focus of the study was on eventual deployment in glaucoma, subjects with nonglaucomatous optic nerve disease were also included. The reason for this was to obtain a diversity of defects to characterize and differentiate a functional vulnerability zone. For the same reason, healthy subjects with no glaucoma or retinal disease, glaucoma suspects, and subjects with preperimetric glaucoma were also included. For the purposes of the study, glaucoma was defined as the presence of characteristic glaucomatous optic nerve head changes (including any or all of: an enlarged cup, neuroretinal rim thinning or retinal nerve fiber layer loss) on clinical examination and imaging, with or without a visual field defect, and with no other ophthalmic or medical explanation for the clinical appearance. Although glaucoma has characteristic visual field defects that are different to those of other neuro-ophthalmic conditions,^18^^,^^19^ the analytic approach described below was not specifically contingent on the presence of a glaucomatous pattern.

### Functional Vulnerability Zone Calculations

**Figure 1:**
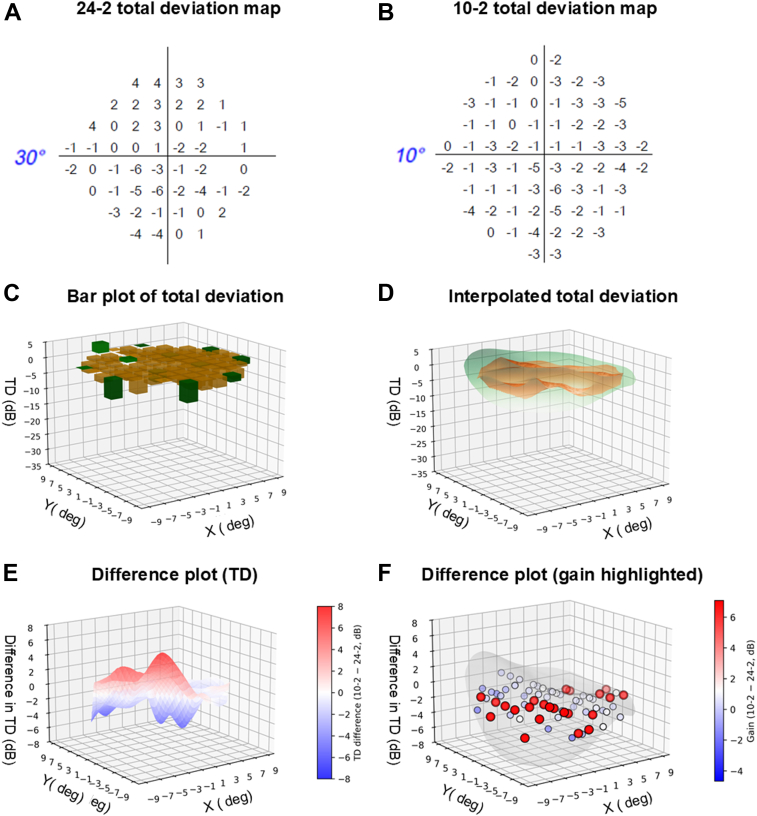
Steps involved in the present study. **A, B,** The total deviation (TD) maps for the 24-2 and 10-2 test grids from the same patient, respectively. For reference, the test radius is shown on the left of the x-axis for the respective grids. **C,** A bar plot extracted from the 24-2 (dark green) and 10-2 (orange) test grids, respectively, with X and Y coordinates representing the Cartesian coordinates of the Humphrey Field Analyzer. **D,** The interpolated surface, which was then used to perform volumetric analysis for 24-2 (green) and 10-2 (orange) test grids, respectively, with an additional 10° radius mask applied. **E,** The difference (10-2 – 24-2) in TD values using an interpolated volume, with a red (positive) result indicating a region where the 10-2 provides additional dynamic range. **F,** The difference in gain using 10-2 test locations compared with interpolated 24-2 test locations. Similar to (**E**), a red (positive) result indicates a region where the 10-2 provides additional dynamic range, with the individually “gained” 10-2 test locations further highlighted with a black outline.

The pipeline for this experiment was a custom script written in Sublime Text and implemented in Python version 3.10. The pipeline entailed the following steps: (1) 10-2 and 24-2 perimetric data extraction and combination, (2) interpolation and volumetric analysis, (3) pointwise gain analysis, (4) principal components analysis (PCAs), (5) regression analysis, and (6) final visualization and GUI construction. Steps 1 to 3 are first detailed below and represent the background methodology for calculating the main end points of perimetric volume and the functional vulnerability zone. Figure 1 shows these steps.

In the first step, portable document format files of 24-2 and 10-2 perimetric data were exported from FORUM (Carl Zeiss Meditec), including: patient ID, date of visit, and time of test (to match 24-2 and 10-2 test results); global indices (mean deviation, pattern standard deviation, and Visual Field Index); false-positive rate; pointwise sensitivity (in decibels); and pointwise TD (in decibels) (Fig 1A, B). These were then manually crosschecked by one of the authors for accuracy. Then, 10-2 and 24-2 data from the same subject were combined into a single comma-separated values file for the next steps.

In the second step, we performed interpolation and volumetric analysis of the combined 10-2 and 24-2 perimetric data. The Cartesian coordinates of the test grid (in degrees) were used as the X and Y values, and the TD results (in dB) were used as the Z values (Fig 1C). Total deviation, rather than sensitivity, was used because it accounts for differences from an age-matched subject within the normative database, effectively representing a normalized result across visual space. Note that although this approach may be better represented by “topography,” we use “volumetric” and “volume” in line with previous work described by Weleber et al.^20^

We defined the regular grid in 1° steps in linear space, which was then interpolated as degrees^2^. The volume was therefore defined using the base area (in degrees^2^) and the TD (in dB). Volume was calculated for the central 10° from fixation. However, because each perimetric test location is covered by a small stimulus area, the TD result was interpolated between points to form a final 3-dimensional structure. A linear interpolation approach was used, as a cubic interpolation tended to significantly overestimate the resultant volume. We purposely chose linear interpolation because it can “bridge” across scotomata, thereby assessing the central question of concordance or discordance between 24-2 and 10-2 test grids, depending on the ground truth state.^15^ In brief, under an ideal scenario, there would be one-to-one concordance between 24-2 and 10-2 test grids, whereby the linear interpolation across 24-2 test locations matches the underlying 10-2 result in terms of sensitivity “volume.” Overestimation by the 24-2 test grid would occur in situations with a relatively deeper or steeper scotoma. Conversely, underestimation of visual field sensitivity “volume” would occur if the 10-2 test grid result was not as deep as that of the interpolated 24-2 test grid. Fundamentally, this represents the “least assumption” version of the functional vulnerability zone in the context of the fixed, rectilinear test grid of the Humphrey Field Analyzer, as linear interpolation does not explicitly fit models across adjacent test locations.

After linear interpolation, we then applied a binary cylindrical mask to the perimetric test area with radius 10°, such that the interpolation that was performed extended only to those areas (Fig 1D). This was the final area used to compute the volume metric.

The difference in volume between the 24-2 and 10-2 test grids was the first outcome measure of interest, essentially representing a global score for the functional vulnerability zone. Mathematically, we defined this as 10-2 – 24-2, such that a “positive” volume difference would indicate that the defect was greater using the 24-2, and thus a greater residual dynamic range for the 10-2 test grid (Fig 1E).

(Equation 1) $$V=A_{cell}\sum_{i=1}^{N_{x}}\sum_{j=1}^{N_{y}}M_{ij}{\hat{z}}_{ij}$$

where ${\hat{z}}_{ij}$ is the interpolated value of z at $\left(x_{i}{,y}_{i}\right)$, $M_{ij}$ is the mask, $A_{cell}$ is ΔxΔy (which is the *x*, *y* distance between test locations) and V is the total visual field volume.

In the third step, we interpolated perimetric data to perform comparisons between 10-2 and 24-2 test grids. Although some test coordinates overlap, many test locations are distinctly tested by each test grid. The volume calculation in step 2 describes a global difference between test grids to highlight the functional vulnerability zone, but we also interpolated sensitivities from the 24-2 at equivalent, real, and measured 10-2 test locations. We referred to this as the “gain” metric with the units based on the absolute number of points. Interpolation was performed using a cubic method to account for the neighboring test locations in the test grid. In contrast to linear interpolation used for global volume, cubic interpolation was used here to model and predict potential 10-2 equivalent sensitivities using known information from the measured 24-2 test locations. A “least assumptions” model of linear interpolation may be suitable for estimating global differences, but is less suited for modeling additional intervening test locations, which was our interest in this step. This is described in more detail in the Discussion.

Simply, “gain” was mathematically defined by the number of clinically measured test locations in which the 10-2 grid had a greater residual dynamic range compared with the interpolated equivalent 24-2 test grid location (Fig 1F). Therefore, such as volume difference, a more “positive” gain indicates a benefit using the 10-2 test grid, as it means there is more sensitivity “remaining.” In this definition of gain, we used a binarized approach of positive or negative gain as a sign-based, rather than magnitude-based, metric. A threshold was not used as it would introduce a potential systematic bias, ultimately favoring the 24-2 test grid by suppressing smaller defects. Suppression of smaller defects would potentially underreport the utility of 10-2 test grid in earlier stages of glaucoma with milder loss, and skew its use toward more advanced stages. Volume differences described in the preceding step already examined magnitude-based differences, which we expected to have more variability because of inherent visual field variation.

To reduce the likelihood of a false-positive functional vulnerability zone identified by isolated, nonclustered regions of deficit, we further refine the criterion by requiring that the volume or gain result favored the 10-2 across ≥3 contiguous 10-2 test locations. Therefore, for example, this meant that an overall volume calculation favoring the use of the 10-2 test grid, which was not supported by ≥3 contiguous points demonstrating the volume difference, was not considered to have a functional vulnerability zone, which aligned with the binarized, nonthresholded approach described above.

In summary, we mathematically defined the main outcome measure, the presence of a functional vulnerability zone, with 2 quantities: (A) difference in TD volume using the same cylinder, and (B) difference in “gain.” Notably, the overall volume represented a “global” metric, whereas “gain” represented localized pointwise differences.

### Predictors of the Presence or Absence of a Functional Vulnerability Zone

Step 4 of the pipeline was regression and PCAs. One of our goals was to identify factors that predict the likelihood of a functional vulnerability zone. We examined this using 2 methods: conventional regression analysis (the “simple” approach), and PCA (the “advanced” approach). The reason for splitting this was for the practicalities of the visualization step (described more below).

Starting with the advanced approach, PCA was used to identify perimetric parameters of interest that could potentially separate results in which a functional vulnerability zone was present or absent. Principal components analysis has been similarly used by our group for perimetric and clinical data.^21^^,^^22^ In the present work, PCA offers the advantage of grouping variables that are potentially covariable or correlated, which is likely to occur in intrasubject perimetric data. The other advantage of performing PCA before the simpler logistic regression analysis is that the loadings of the principal components provide insights on the relative contribution of each perimetric parameter.^15^

In brief, key 24-2 test grid parameters were first normalized to produce Z-scores so they could subsequently be combined. The parameters included: mean deviation; pattern standard deviation; Visual Field Index; central (12 points) sensitivity volume and TD volume; central interpolated cylindrical (10° from fixation) sensitivity volume and TD volume; and number test locations with TD probability scores of *P* < 5%, *P* < 2%, *P* < 1%, or *P* < 0.1%. We used TD, rather than pattern deviation, because pattern deviation results are modulated by the subject’s Hill of Vision. As the 24-2 has many more peripheral test locations that may influence the Hill of Vision than the 10-2 test grid, we aimed to reduce their contribution to comparisons between central test locations.

Because mean deviation and TD probability scores were notably nonlinear and bounded in their distributions, we applied additional transformations to these factors before Z-score conversion as follows: for mean deviation, shift-log (to reduce left skewness); and for TD probability scores, log1p transformation (to reduce zero compression). Because of the ceiling effect exhibited by the Visual Field Index, this was not included in the PCA. Only factors with loadings of >0.35 were retained. Such factors were then considered significant and used in the simple logistic regression analysis below. We used the top 2 principal components (PC1 and PC2) as predictors for each of the outcomes using logistic regression analysis.

For the simplified approach, the predictors included the 24-2 parameters that were found to be significant in the PCA. The reason for having a simplified approach is to improve the interpretability and user-friendliness of the final visualization step.

In step 5, we used the logistic regression analysis to determine the final contributions of each parameter identified to be significant factors in step 4. For the simplified approach, the 2 features with the highest factor loading identified in PCA were taken as the predictor matrix, and the binarized target was the benefit from the 10-2 test grid, that is, the presence of a functional vulnerability zone. For the advanced approach, PC1 and PC2 were used in the predictor matrix. Because some subjects contributed >1 eye or data point to the data set, we used generalized estimating equations for clustering (equation 2).

(Equation 2) $$\text{logit}\left(p_{ij}\right)=\beta_{0}+\beta_{1}{\tilde{X}}_{1,ij}+\beta_{2}{\tilde{X}}_{2,ij}$$

The output model coefficients were saved as a pickle file for use in the visualization and GUI step.

### Visualization and GUI

In the final step of the pipeline, we developed a prototype GUI as a basis for future clinical use. The GUI was designed to allow inputs at both basic and advanced levels, representing inputs from the simplified and advanced PCA models from step 4, respectively. The inputs would represent prospective data for comparison against the models, including all the necessary calculation steps, including data standardization and probability prediction. The output would be a percentage-based likelihood of a measurable benefit from the 10-2 using the outcome measures described above.

### Statistical Analysis

Descriptive statistics were used to summarize the basic demographic and clinical characteristics of the cohort, with parametric or nonparametric statistical analyses applied as appropriate. Other statistical tests have been described above as per the pipeline steps.

Additionally, in the subset of subjects with repeated measures (i.e., the same eye but on different days), we examined the stability of the binarized functional vulnerability zone outcome between visits. Because there were an unequal number of results per subject and per eye, and because visual field tests may undergo longitudinal change, we used state-transition and stability analyses. This analysis returns a stability index and absorbing tendency. Stability index is mathematically defined as 1 – (number of state changes/number of visits – 1). A stability of 1.0 represents perfect stability, and 0.0 represents alternating states and substantial noise. Absorbing tendency represents directional consistency, conceptually defined as if the outcome changes (such as from zone absent to zone present), whether it remains as the new outcome. This approach offers advantages of being able to model outcomes beyond pairwise comparisons (as per Cohen’s kappa or Gwet’s AC1), and incorporates temporal information in situations where progression and changes to variability are likely to occur longitudinally.^23^

A *P* < 0.05 was considered statistically significant. Visualization of the data was performed using GraphPad Prism version 9.4.0 (GraphPad Software).

## Results

### Baseline Characteristics of the Cohort

The baseline characteristics of the cohort of 160 subjects are reported in Table 1 (some subjects contributed only 1 eye, whereas others contributed 2). The total number of eyes with a pair of 24-2 and 10-2 test grid results analyzed was 626. Approximately half of the cohort had been diagnosed with glaucoma, and almost one-third were glaucoma suspects. The remaining diagnoses were approximately equally divided into nonglaucomatous optic nerve disease and normality. Although we recognize that some studies have suggested clinical phenotypic differences in visual field defects, the purpose of our study was to capture a breadth of potential defects to understand the functional vulnerability zone. Therefore, the diagnostic groups were not further differentiated.

**Table 1:** Baseline Demographic and Distribution of Clinical Diagnoses for the Present Cohort

	Right Eye (N = 132)	Left Eye (N = 138)	*P* Value
Age (yrs)	62.5 (10.7)	63.4 (10.8)	0.5754
Gender (M:F, n, %)	70:62 (53%:47%)	78:60 (56.5%:43.5%)	0.792
Days between 24-2 and 10-2 test	0 (IQR, 0–0; full range, 0–12)	0 (IQR, 0–0; full range, 0–12)	0.9791
IOP (mmHg)	16.6 (4.3)	16.6 (4.3)	0.8911
CCT (μm)	548.4 (35.1)	551.7 (32.4)	0.4893
Mean deviation 24-2	–1.29 (IQR, –2.87 to –0.06; full range, –10.34 to 3.79)	–1.77 (IQR, –3.37 to –0.63; full range, –16.06 to 1.38)	0.565
Mean deviation 10-2	–1.34 (IQR, –3.00 to –0.20; full range, –6.17 to 1.40)	–1.67 (IQR, –3.11 to –0.54; full range, –14.51 to 1.90)	0.7157
Glaucoma∗	66 (50%)	73 (52.9%)	0.6447
Glaucoma suspect†	41 (31.1%)	34 (24.6%)	
Nonglaucomatous optic neuropathy	10 (7.6%)	14 (10.1%)	
Normal	15 (11.4%)	17 (12.3%)	

CCT = central corneal thickness; F = female; IOP = intraocular pressure; IQR = interquartile range; M = male.

Age and self-identified gender are reported per subject, with clinical parameters and diagnoses separated by eye.

∗ Primary open-angle glaucoma, normal tension glaucoma, secondary glaucoma, and angle-closure glaucoma.

† Primary open-angle glaucoma suspect, ocular hypertension, pigment dispersion syndrome, and pseudoexfoliation.

### Functional Vulnerability Zone Calculations

**Figure 2:**
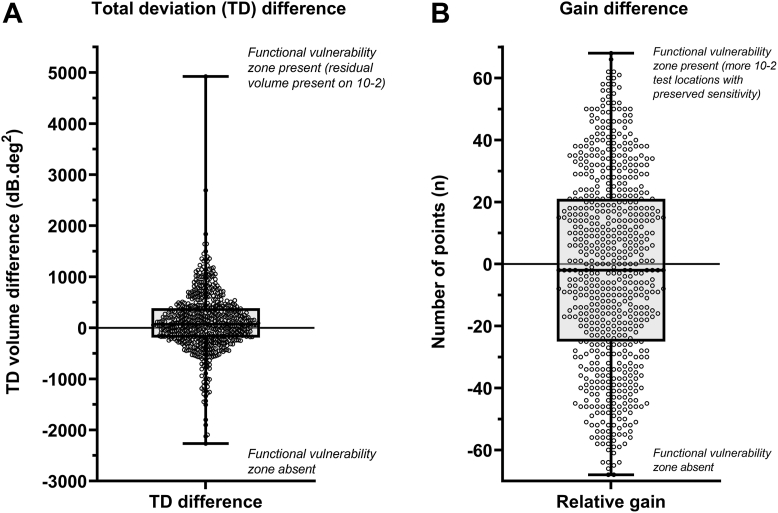
**A,** Difference in total deviation (TD) volume (10-2 – 24-2) after volumetric analysis. A more positive result indicates a functional vulnerability zone present (with residual volume present on the 10-2 test grid). **B,** Difference in gain (10-2 – 24-2 test locations). A more positive result indicates a functional vulnerability zone being present, whereby the 10-2 test grid had more test locations with residual sensitivity compared with the interpolated 24-2 test grid. In both panels, each open circle represents an individual subject’s visual field result, and the box and whisker plots indicate the median, interquartile range, and full range.

The median difference in total volume across the cohort was 70.5 dB.deg^2^ (interquartile range [IQR], –191.1 to 386.0 dB.deg^2^), indicating that most subjects had a calculable functional vulnerability zone suggesting a potential benefit of using the 10-2 test grid (*P* < 0.0001 on a 1-sample *t* test) (Fig 2) The median difference in gain in test locations was –2 (IQR, –25 to 21 points) (Fig 2). A 1-sample *t*-test was not statistically significant (*P* = 0.1874).

### Factors Related to Functional Vulnerability Zone Calculations

**Figure 3:**
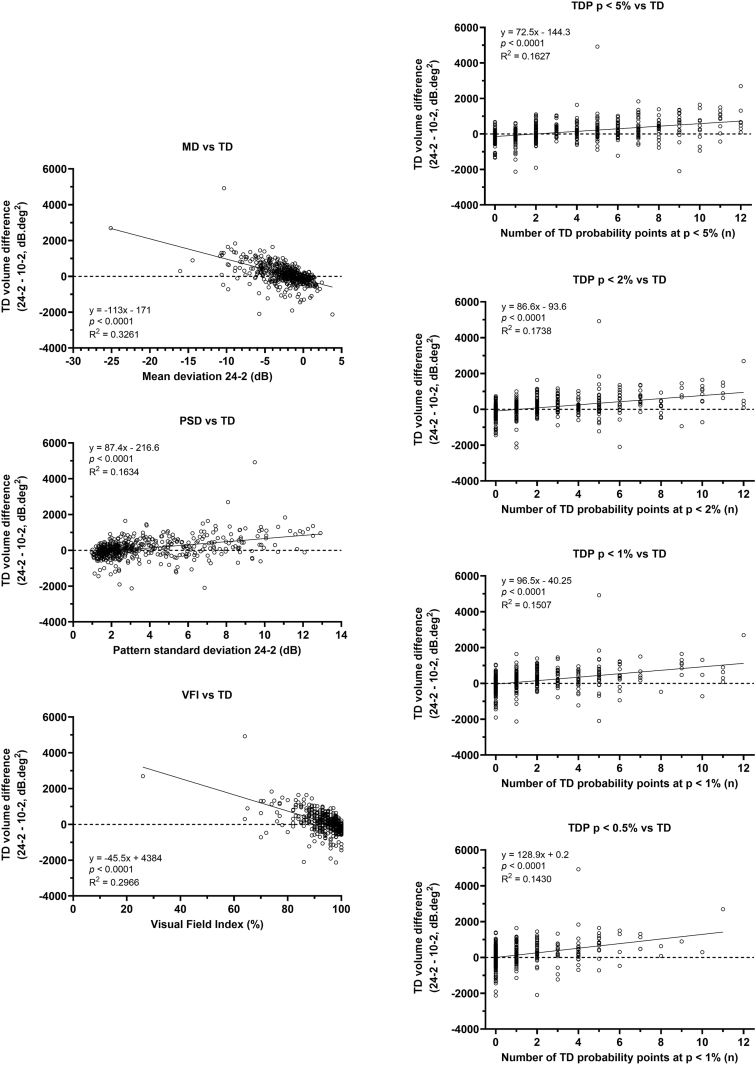
Linear regression analysis of key perimetric parameters and the difference in total deviation volume calculated using volumetric analysis. Each datum point indicates the result from one subject, with the regression analysis results shown in the inset MD = mean deviation; PSD = pattern standard deviation; TD = total deviation; TDP = total deviation probability; VFI = Visual Field Index.

**Figure 4:**
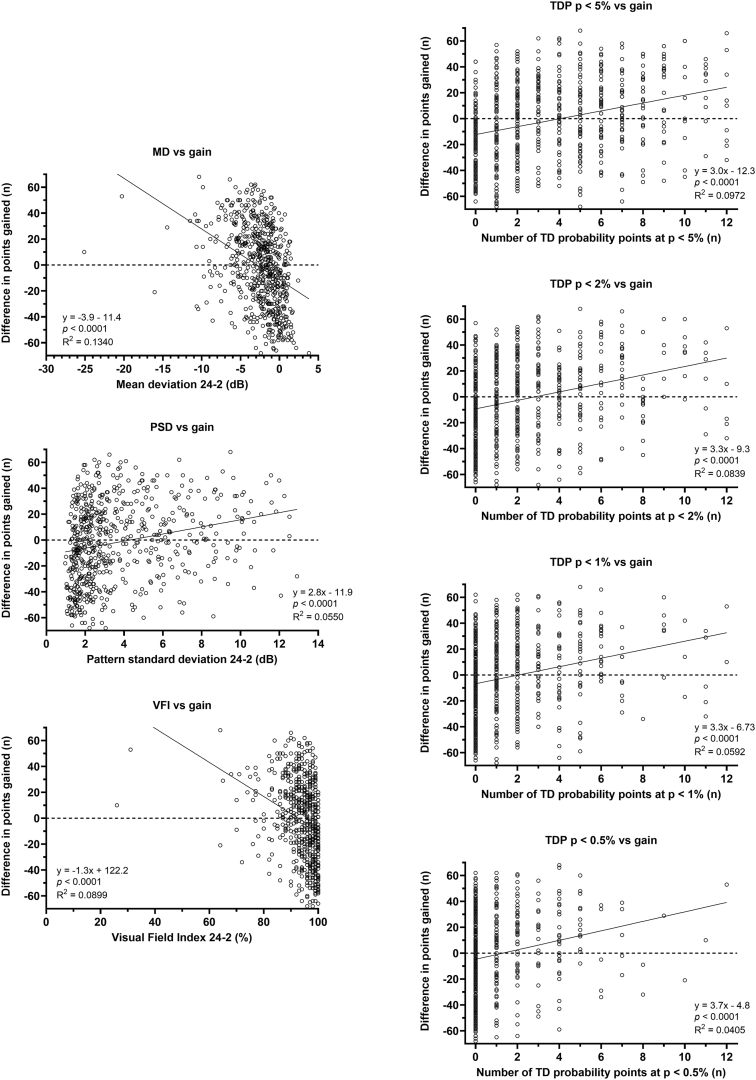
Linear regression analysis of key perimetric parameters and difference in gain (number of 10-2 locations – number of interpolated 24-2 locations with residual sensitivity) calculated using volumetric analysis. Each datum point indicates the result for one subject, with the regression analysis results shown in the inset. MD = mean deviation; PSD = pattern standard deviation; TD = total deviation; TDP = total deviation probability; VFI = Visual Field Index.

Linear regression analyses were performed using the 2 functional vulnerability zone outcomes (TD volume difference, Fig 3; and gain, Fig 4) as dependent variables, and mean deviation, Visual Field Index, pattern standard deviation, and number of TD probability plot locations (out of the 12 central 24-2 test locations) significant at the *P* < 5%, *P* < 2%, *P* < 1%, and *P* < 0.5% levels. There was a statistically significant result for regression analysis when using each factor in both the TD difference and gain metrics (all *P* < 0.0001). Coefficients of determination were higher when examining TD difference (range, 0.1430–0.3261) compared with gain metrics (range, 0.0405–0.1340). This was likely due to a wide distribution of gain results at the “ceilings” of each parameter. The highest coefficient of determination was mean deviation versus TD difference (0.3261), but was nonetheless relatively low, suggesting that the 24-2 and 10-2 test grids offer unique information regarding the status of the central visual field.

### Visualization Using PCA

Using inputs from the 24-2 test grid (mean deviation, Visual Field Index, pattern standard deviation, and number of TD probability points at the *P* < 5%, *P* < 2%, *P* < 1%, and *P* < 0.5% levels), we performed PCA to identify correlations between parameters that could be used to separate subjects with and without a functional vulnerability zone. The resultant loadings, filtered to only include those with loadings >0.35, are shown in Table 2. Only 5 factors remained significant, with mean deviation and TD probability scores at *P* < 1% contributing to PC1 and pattern standard deviation, TD probability scores at *P* < 5% and *P* < 2% contributing to PC2. Principal component 1 accounted for 69.3% of the variance, and P2 accounted for 10.4%, with both their contributions totaling 79.7% for the model.

**Table 2:** Loadings of Significant Factors for Principal Components 1 and 2 after Filtering

	PC1 (69.3%)	PC2 (10.4%)
MD 24-2	0.3599	
24-2 TD volume (calculated using a cylindrical mask within the central 10°)		0.4888
24-2 TD volume (calculated using central 12 points only)		0.4580
TD probability plot points at *P* < 2% within the central 12 points	–0.3536	0.3791
TD probability plot points at *P* < 1% within the central 12 points		0.3881

MD = mean deviation; PC = principal component; TD = total deviation.

The variance accounted for by each PC is shown in brackets. The empty cells indicate no loadings for that factor for the principal component.

Although the central volumetric parameters were also found to be significant on PCA, we chose mean deviation from PC1, and the number of TD probability scores at *P* < 1% (among the central 12 24-2 test grid test locations) from PC2 to represent the simplified model. These were chosen because both represent the highest, clinically interpretable metrics easily extractable from the perimetric printout (as the volume parameter requires further computation), facilitating clinical deployment.

The PCA scores are summarized in Table 2. In brief, the magnitude of PC1, which accounts for most of the variance of the model, decreased with worsening 24-2 mean deviation and an increasing number of TD probability map points at the *P* < 2% and *P* < 1% levels, indicating a worse 24-2 visual field result. Because of the nature of PCA, PC2 describes the rest of the variance that was not explained by PC1. In this case, PC2 describes the spatial extent of defects, translating to deeper depressions being described by a high PC2 (high TD probability points and small defect volume) and shallow diffuse losses being described by a low PC2.

**Figure 5:**
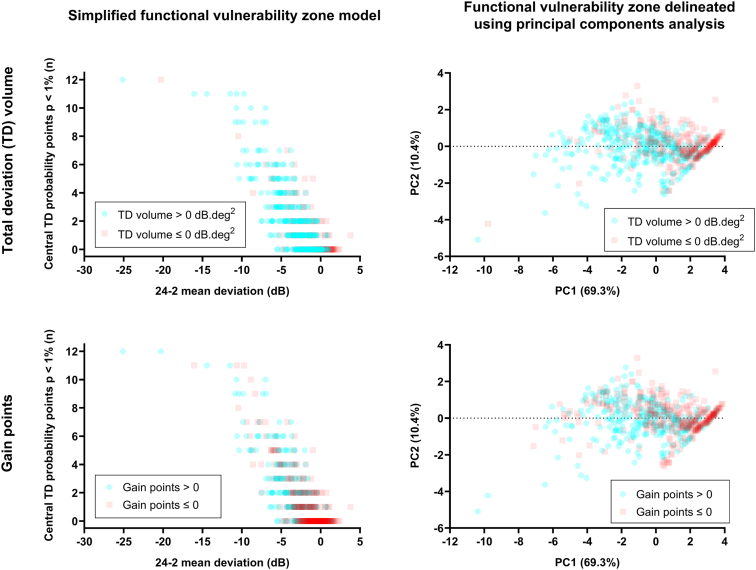
Left column: The simplified functional vulnerability zone model showing the distribution of number of total deviation (TD) points at the *P* < 1% within the central 24-2 visual field as a function of 24-2 mean deviation result. The presence of a functional vulnerability zone is represented by a TD volume difference >0 dB.deg^2^ (teal), with the absence shown by a result ≤0 dB.deg^2^ (red). Right column: The functional vulnerability zone delineated using principal components analysis, plotting principal component (PC)2 as a function of PC1 (with the amount of variance accounted for by each in brackets). The color-coded result is the same as the left column.

The resultant simplified and PCA models with data points separated by volume and gain thresholds are shown in Figure 5. In the simplified model, a worsening mean deviation and an increased number of TD probability points at the *P* < 1% level increased the likelihood of a functional vulnerability zone being present (teal circles) compared with absent (red squares). When using PCA, a more negative PC1 result (i.e., a worse mean deviation result and more test locations on the TD probability plan at the *P* < 2% or *P* < 1% levels) favored a functional vulnerability zone. Principal component 2 contributed less to this relationship, but in general, a more negative PC2 result also favored a functional vulnerability zone. For both simplified and PCA models, the separation between a functional vulnerability zone being present or absent was more obvious when using the gain metric, rather than the TD volume calculation.

### Stability Index and Absorbing Proportion

There were 355 instances of repeated measurements across 151 eyes. The state transitions (whether functional vulnerability zone absent [0] or present [1] and their stability between visits) for volume and gain approaches are summarized in Table 3. Median stability was calculated per eye, and was 0.67 (IQR, 0–1) and 0.75 (IQR, 0.33–1.00) for binarized volume and gain, respectively. The absorbing proportions were 0.76 and 0.83 for binarized volume and gain, respectively, though, unlike kappa, there are no widely accepted qualitative descriptors of these magnitudes.

**Table 3:** Number and Proportion of Occurrences of Each State Transition (0 = Functional Vulnerability Zone Absent; 1 = Functional Vulnerability Zone Present) for Binarized Volume and Binarized Gain Conditions

State Transition	Binarized Volume	Binarized Gain
0 → 0	111 (31.3%)	34 (9.6%)
0 → 1	81 (22.8%)	69 (19.4%)
1 → 0	68 (19.1%)	44 (12.4%)
1 → 1	95 (26.8%)	208 (58.6%)

### Graphic User Interface and Case Examples

Using the data above, we created a prototype GUI that could be used to examine prospective case examples. In brief, the GUI contains input boxes for mean deviation, number of TD probability (*P* < 2%) points centrally, PC1 and PC2 scores. A drop down at the top allows the user to select whether to examine the TD difference or gain difference based on volumetric analysis. There is a checkbox for “Advanced mode” (which uses the PC results). Otherwise, “Basic mode” involving only mean deviation and TD probability points is used to calculate the probability.

A “Load PDF and Predict” button is available if a suitable file can be uploaded to the application (portable document format exported from FORUM), which will automatically read and input the relevant parameters. A “Predict” button is available after manual user input of values. A text box shows a brief statement on the likelihood of benefit of using a 10-2 test grid, given these 24-2 test grid results, with a yellow highlighted box indicating a likely equivocal result.

The plotted area below the inputs and outputs shows the range for potential inputs. The blue to red zone represents the probability of benefit of using a 10-2 test grid. There is a yellow band abutting the black dashed line (of unity), which nominally represents probability values of 0.35 to 0.65 (0.15 either side of 0.5). Thus, a darker blue or a darker red represents a progressively lower and higher likelihood of a benefit of using the 10-2 test grid, respectively. In the upper right and lower left regions of the plot, there are 2 additional grayed regions. These regions indicate areas where sampling was nonexistent or sparse in the present cohort, and also clinically represent low likelihood scenarios. For example, the upper right region corresponds to a very positive mean deviation score, but the presence of a high number of TD abnormalities. This is unlikely to be true pathology and is more likely representative of an artifact. A similar principle is applied to the lower left region, represented by a very negative mean deviation value and a smaller number of affected central test locations.

**Figure 6:**
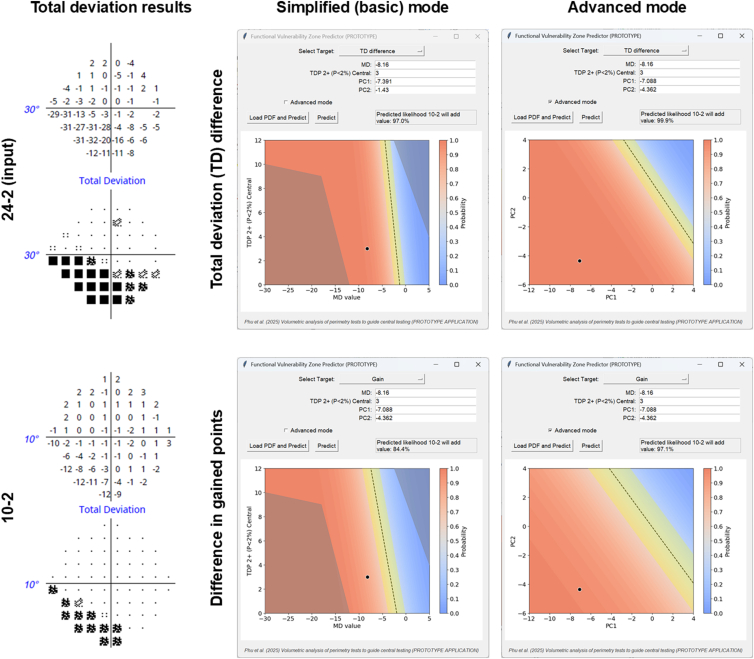
A representative case of a subject with glaucoma, with their data input into the graphic user interface (GUI). The 24-2 test result is the input (total deviation, numerical, and probability scores shown in the top left). For reference, their 10-2 test result is shown on the bottom right. The functional vulnerability zone GUI is shown in the right hand columns, with an example of basic and advanced mode outputs demonstrated.

One representative case is shown in Figure 6, with the different GUI display options shown in the middle and right columns. Six more representative case examples of the application of this GUI in subjects not included in the development cohort are shown and, for brevity, more extensively discussed in the Figures S1–S6 (available at www.ophthalmologyscience.org). In brief, after the inputs are provided, the output figure highlights regions with different probabilities of 10-2 test grid utility (as indicated by the color bar: redder results indicate a higher probability). The prospective patient is shown in the black circle. In Figure 6, the position of the black circle in both the basic and advanced modes indicates a relatively high likelihood of 10-2 utility.

## Discussion

In the present study, we used volumetric analysis of TD visual field results to characterize a functional vulnerability zone, which aims to identify situations where a 10-2 test grid provides more clinical information than the 24-2 test grid for progression analysis. With the goal of providing clinically relevant end-user benefit, our model suggested mean deviation and the number of TD probability points at the *P* < 2% (within the central 12 test locations of the 24-2 test grid), beginning with at least a mean deviation of –5.50 dB with at least one reduced test location centrally, as the main drivers of the presence of a functional vulnerability zone (probability >90%). Principal components analysis provided further insights in separating between those with and without a functional vulnerability zone. These results culminated in the creation of a prototype GUI for characterizing prospective patients.

### Characterizing the Functional Vulnerability Zone

We previously introduced and discussed the underlying theory of the functional vulnerability zone in the context of visual field sensitivity outputs.^15^ In brief, the functional vulnerability zone represents a clinically detectable and relevant region in which residual visual function can be meaningfully measured. Because there is discordance between the relative density of ganglion cells subserving central visual function and the sparse sampling of the 24-2 test grid in the same region, volumetric analysis may highlight differences between sparsely and coarsely sampled test regions of interest.

In the present study, we used TD instead of sensitivity. Total deviation provides an age-corrected indication of a defect and is used for identifying significant visual field loss. The present results were consistent with our previous work on sensitivity.^15^ We found similar relationships between volumetric analysis obtained using TD and key perimetric indices, such as mean deviation.

The functional vulnerability zone represents a functional approach to assessing the need for targeted central visual field testing. This is notably different to using structural imaging to guide visual field testing, which has been illustrated in previous studies.^24^^,^^25^ An advantage of structurally guided testing is potentially achieving structure-function concordance and thus more robust evidence for clinical decision-making. It may also lead to a more efficient test approach by assessing high-yield locations. Simultaneously, such approaches intrinsically rely on a robust relationship in the first instance—a relationship known to be highly variable, especially with increasing substrate loss.^26^^,^^27^ As a functional outcome, the functional vulnerability zone is also different from the macular vulnerability zone proposed by Hood et al,^28^ which is based on empirical evidence of commonly affected anatomical regions.

We approached this clinical problem differently. Foremost, the goal of the functional vulnerability zone is to identify candidates for whom using the 10-2 test grid may aid in progression analysis, as it highlights residual visual function that can be clinically assessed. Although structural data may be useful for identifying regions of interest to confirm the presence of a functional deficit, there remain unknowns regarding the structure-function relationship for characterizing dynamic range as described in the present work. This relationship is also notably poorer at early and later stages of pathological loss because of the limitations of the respective instruments.^26^^,^^27^ This manifests clinically as differences in progression detection using structural and functional testing at different stages of glaucoma.^29^^,^^30^ Because the main fundamental difference between 24-2 and 10-2 test grids is their test density, the functional vulnerability zone represents a more meaningful functional approach to assessing the utility of a denser test grid.

A previous study by Weleber et al^20^ used thin plate spline interpolation for characterizing the Hill of Vision or volume or the visual field. There are several differences between thin plate spline interpolation and linear and cubic approaches used in the present study. Thin plate spline interpolation treats the visual field test grid as a global problem and aims to optimize the result using that perspective. Thus, using thin plate spline, points are considered globally across all sampled test locations. This contrasts with cubic interpolation, which examines neighboring points alone, with each region being described by its own curved surface. In the present study, the functional vulnerability zone is assumed to have local information that could be gleaned by using the 10-2 test grid. This could be facilitated by cubic interpolation, especially in the setting of milder glaucomatous defects that would be highly localized, rather than global. Another difference between the present work and that of Weleber et al^20^ is that we used the rectilinear grid of the Humphrey Field Analyzer, rather than the radial grid of the Octopus perimeter. Cubic interpolation could offer advantages over thin plate spline in view of the Cartesian coordinates and grid topology.

### Comparison with Hybrid or Custom Models

A current hybrid visual field test grid available on the Humphrey Field Analyzer is the 24-2C, which adds an additional 10 test locations within the central 10° from fixation.^6^^,^^31^ This was not collected in the present study. Although it seeks to address the relatively sparse test density of the 24-2 test grid, only modest benefits have been shown in the detection or confirmation of central visual field defects.^6^^,^^31^ The goal of “detection” or “confirmation” of defects is notably different from the clinical question in the present study, which was monitoring for progression through optimizing dynamic range.^1^ There have been no long-term studies examining the role of the 24-2C in longitudinal analysis, which was the central research question in the present work. A future study could apply the same type of volumetric analysis to the 24-2C and compare it to both 24-2 and 10-2 test grids. We refrained from performing a subanalysis of the 24-2C test locations within the 10-2 test grid because of the number of assumptions required for comparison. However, given that only 10 additional test locations are provided in the 24-2C, we expected that it would not likely provide a significant benefit over the 10-2 test grid with respect to pointwise dynamic range.

There exist novel approaches that use existing perimetric results, such as from a relatively sparse 24-2 test grid, or de novo defect mapping to procedurally add more test locations to further characterize a progressive scotoma, thereby enhancing the ability to monitor visual field defects.^32^^,^^33^ These algorithms are advantageous in that they permit monitoring of the peripheral visual field in addition to a tailored approach to central testing. It is likely that there would be eventual convergence upon a 10-2 (or similar) test grid in more advanced stages of glaucoma, beginning with high-yield test locations of interest, as alluded to by Turpin et al.^34^ Both customized, expanding visual field models and the functional vulnerability zone approach represent data-driven methods for guiding additional central visual field testing. The proposed advantage of the functional vulnerability zone is its characterization of the pretest probability of deploying the 10-2 test grid using an established clinical test that has been extensively studied for progression analysis in glaucoma.

### Limitations

The present work was a cross-sectional study and proposes a prototype GUI for eventual clinical deployment. This represents the first phase in the investigation and requires extensive validation with longitudinal data and cost-benefit analysis. Most notably, the question of the optimal volumetric difference—TD difference or gain—for characterizing the functional vulnerability zone needs to be addressed. Furthermore, as a data-driven approach, the conclusions of the present work are based on our specific cohort, and external validation in other clinical contexts is required.

We compared 24-2 and 10-2 test results obtained using the Swedish Interactive Thresholding Algorithm (SITA)-Faster and SITA-Fast test algorithms, respectively. Although derived from the same family of algorithms, there are inherent differences as previously described.^35^ Importantly, SITA-Faster has been shown to return slightly higher sensitivity outputs compared with older SITA versions, and may also return higher sensitivities in the context of significant defects.^36^ To assess this in the present study, we compared sensitivities across the 4 test locations mutually tested across 24-2 and 10-2 test grids (3° horizontally and vertically from fixation). Interestingly, the 24-2 SITA-Faster results were, on average, 0.5 dB (standard deviation 3.5 dB) lower than the corresponding 10-2 SITA-Fast results. The median and interquartile range differences were 1 dB and –1 to 2 dB, respectively. This was statistically significant at *P* < 0.0001, but was clinically small.

Similarly, we recognize that the use of TD, rather than sensitivity, might be affected by underlying normative databases that differ from those of the SITA-Fast and Faster algorithms. This is a limitation of the study. The first iteration of the functional vulnerability zone used sensitivity, which has its own disadvantage of not incorporating age into its output measurement, and thus, the potential significance of relative sensitivity deficit may be difficult to evaluate. Nonetheless, our goal was to compare the current fastest iterations of the 24-2 and 10-2 available in clinical practice.

As previously mentioned, the volumetric approach applied to 24-2 data is inherently an estimate of the subject’s Hill of Vision and, by nature, is limited by interpolation of a coarse area. This is fully intentional and reflects an imperfect approach to clinical visual field testing. Thus, the concept of the functional vulnerability zone assumes the utility of the 10-2 test grid based on imperfect and often poorly correlated 24-2 test results. Nonetheless, the goal is to use a data-driven approach to complement the 24-2 examination.

We did not collect structural data for this study, and thus, structure-function comparisons were not available. The concordance—or discordance—between structure and function and its relation to the 10-2 test grid utility could be studied in the future. We have previously noted that the functional vulnerability zone represents a subjective perimetric outcome,^15^ and is not the same as the macular vulnerability zone described in anatomical studies.^37^

The goal of the study was to develop an end-user GUI that has clinical meaning. Therefore, we focused on high-yield visual field parameters, with the simple model defined by 2 parameters easily appreciable clinically. The disadvantage, as noted above, is that 2 parameters alone do not fully account for the functional vulnerability zone. The PCA results were more complex and likely provide a more nuanced view of the functional vulnerability zone but are more challenging to clinically interpret. Although users have both options, the user must still exercise clinical judgment in interpreting the output result in the context of these assumptions.

### Conclusions

We provide a refined version of the functional vulnerability zone model to provide guidance for deploying the 10-2 test grid for the explicit purpose of providing additional clinical information for progression analysis. We also demonstrate a prototype GUI with a future goal of clinical deployment.
