# Supplementary material for: Volumetric Analysis of Perimetry Tests to Guide Central Testing: The Functional Vulnerability Zone
Source: Ophthalmol Sci. 2026 Mar 12;6(6):101152. doi: 10.1016/j.xops.2026.101152 (PMC13126495; doi:10.1016/j.xops.2026.101152)
Supplement: Figures S1–S6 [file mmc1.docx]

**Supplementary material: Volumetric analysis of perimetry tests to guide central testing****: the functional vulnerability zone**

Jack Phu^1,2^, Henrietta Wang^1^, Michael Kalloniatis^1,2,3^

1. School of Optometry and Vision Science, University of New South Wales, Kensington, New South Wales, Australia
2. University of Houston College of Optometry, Houston, TX
3. School of Medicine (Optometry), Deakin University, Waurn Ponds, Victoria, Australia

Number of Figures: 6 Number of Tables: 2

Corresponding Author: Jack Phu

Address for reprints: School of Optometry and Vision Science, Gate 14 Barker Street Rupert Myers Building South Wing, University of New South Wales Sydney 2052, New South Wales, Australia

Email: [jack.phu@unsw.edu.au](mailto:jack.phu@unsw.edu.au)

*Keywords*: visual fields; perimetry; standard automated perimetry; 24-2; frontloaded

Financial support: The work was supported, in part, by an NHMRC Ideas Grant to MK and JP (1186915). The funding organisation had no role in the design or conduct of this research.

Conflict of interest: No conflicting relationship exists for any author.

Running head: Intrinsic variability in perimetry

Supplementary Figures

Each Supplementary Figure below represents an example case drawn from the glaucoma service of Centre for Eye Health (University of New South Wales), but who had not been included in the development of the model in the present study (i.e. naïve patients). Each Figure is similarly arranged: the functional vulnerability zone GUI is shown on the left, the 24-2 total deviation results (numerical and probability maps) in the centre, and the 10-2 total deviation results on the right.

An explanation of the GUI is provided in the main text.

The purpose of these illustrative cases is to show a diversity of possible outcomes output by the GUI. For cases with probabilities of 10-2 test grid utility of >0.65 or <0.35, the probabilities are reported in the grey box. For those with an equivocal probability (nominally defined as 0.35-0.65), the probabilities are not shown, and it is highlighted in yellow.

Examples of both basic and advanced modes are shown, along the two main surrogates for the functional vulnerability zone: total deviation difference and gain.

A brief description of the case is also provided in each figure caption.


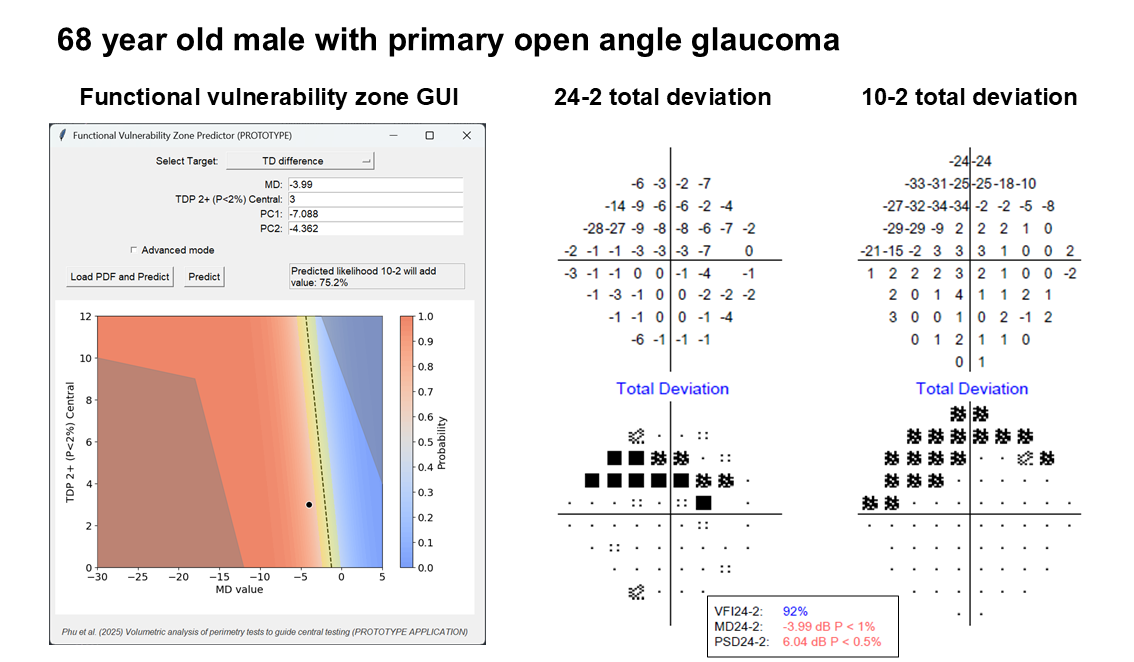


S1: Case of a 68 year old male with primary open angle glaucoma in the right eye. There are three central 24-2 test locations with highly statistically significant sensitivity reduction (*p* < 0.005), but the mean deviation result is only -3.99 dB. The likelihood of 10-2 utility is close to the border of equivocal probability.


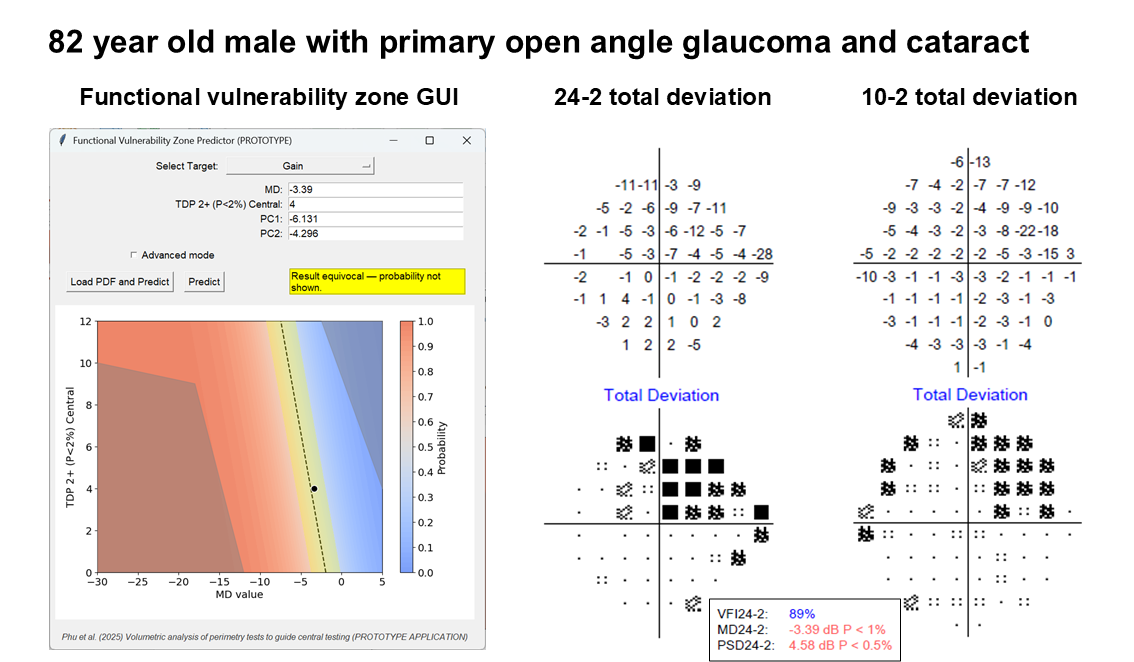


S2: Case of a 82 year old male with primary open angle glaucoma in the left eye. There are four central 24-2 test locations with highly statistically significant sensitivity reduction. In contrast to the case presented in Supplementary Figure 1, the mean deviation result is less severe and with a greater number of total deviation probability points of statistical significance. The likelihood of 10-2 utility is therefore within the border of equivocal probability.


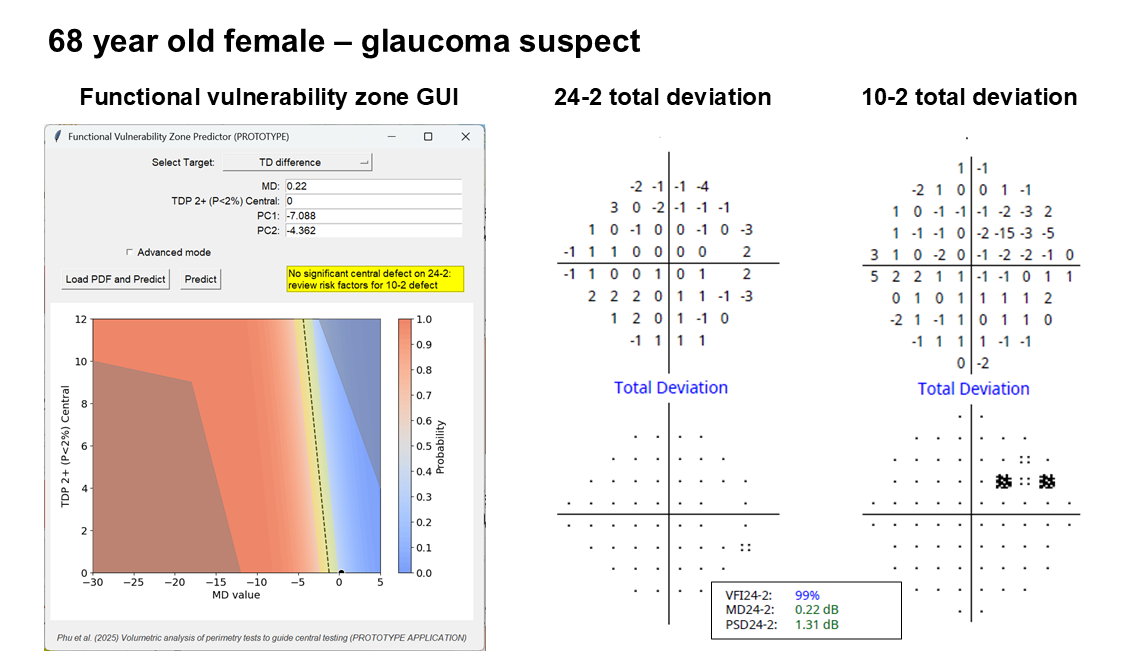


S3: Case of a 68 year old female with glaucoma suspect status in the right eye. There are no central 24-2 visual field defects and mean deviation is also not statistically significantly reduced. However, under some circumstances, the 24-2 may miss a central visual field defect; hence, the suggestion to review other risk factors.


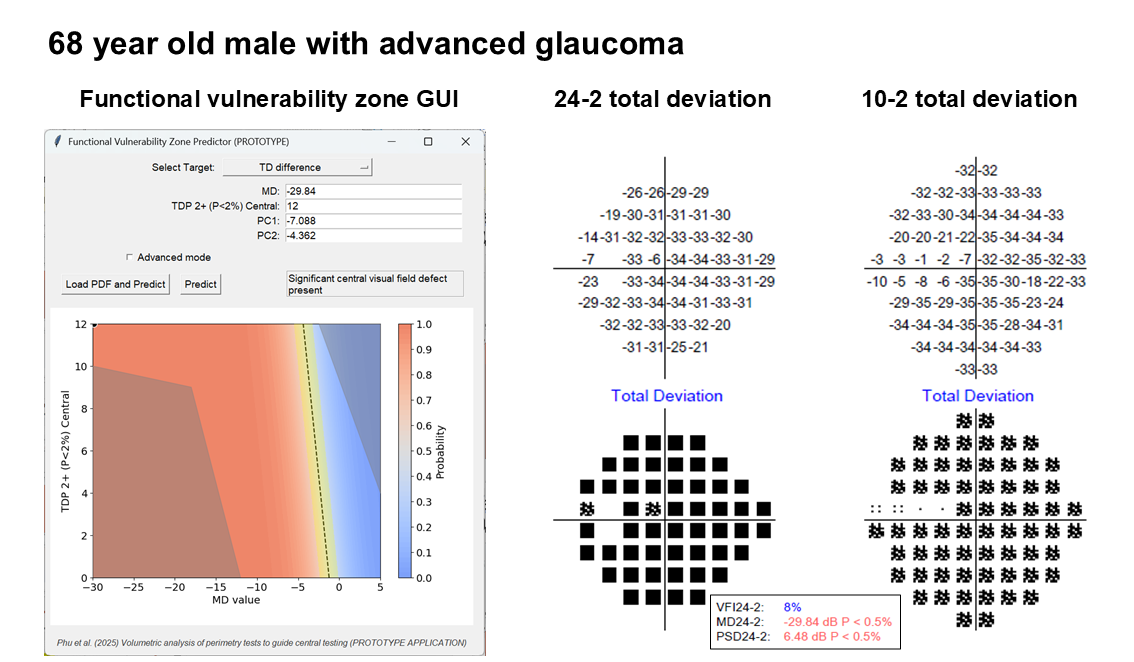


S4: Case of a 68 year old male with advanced glaucoma in the left eye. All central 24-2 test locations are statistically significantly reduced, with a profound loss on 24-2 mean deviation. The outcome clearly indicates a significant central visual field defect. Whilst this means that a 10-2 test grid may have utility, it is also possible that it is also profoundly reduced, with limited clinical use.


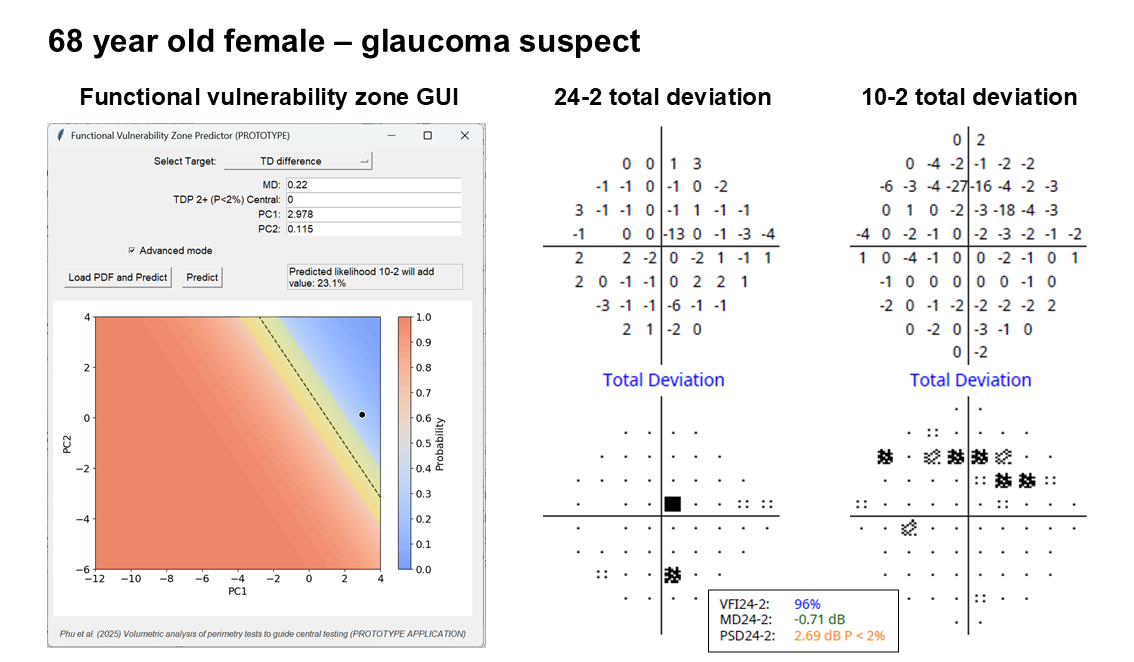


S5: Case of a 68 year old female with glaucoma suspect status in the left eye. This is an example of advanced mode, where the PDF reader examines the entire 24-2 test result and computes principal components 1 and 2 before plotting them on the figure. Surrogates for this would be the 24-2 mean deviation and the number of 24-2 total deviation probability points with different levels of statistical significance. There is only one central 24-2 test location with statistically significant deficit. The mean deviation is also not statistically significantly reduced. Therefore, the likelihood of 10-2 providing additional benefit is low (23.1%).


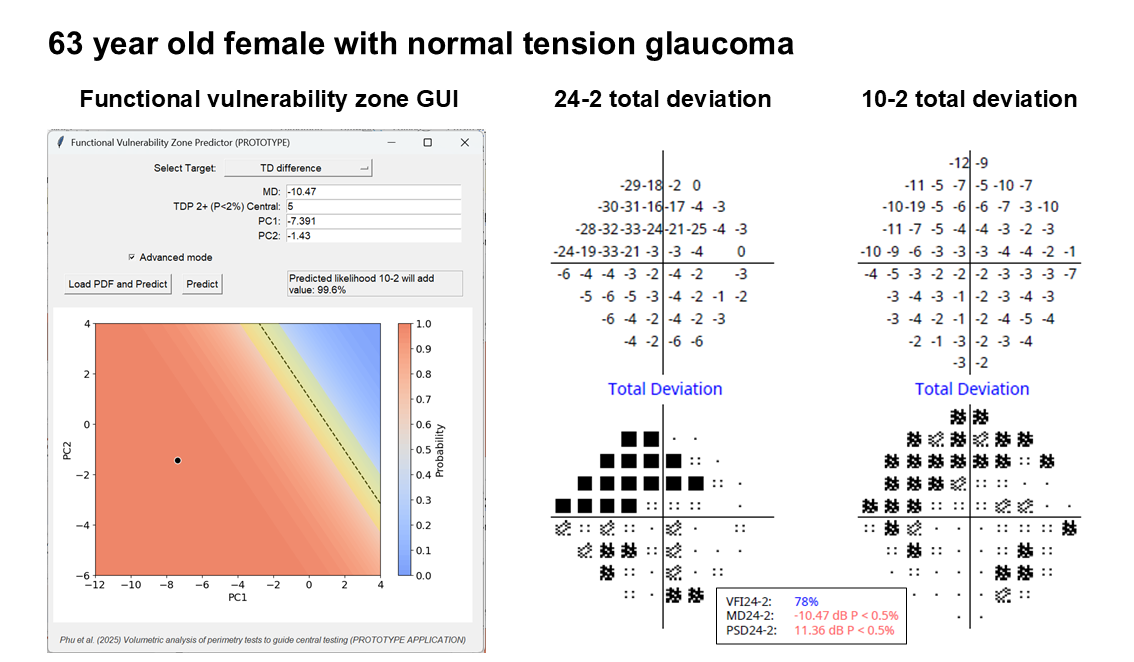


S6: Case of a 63 year old female with normal tension glaucoma in the right eye. This is an example of advanced mode, where the PDF reader examines the totality of the 24-2 test result and obtains values for principal components 1 and 2 before plotting on the figure. Surrogates for this would be the 24-2 mean deviation and the number of 24-2 total deviation probability points at different levels of statistical significance. In this case, there are 5 test locations at the *p* < 0.02 or lower level, and a mean deviation of -10.47 dB, suggesting advanced central loss, and thus a higher probability of 10-2 test grid utility.
